# Interacting with IoT Data Spaces Using LLMs and the Model Context Protocol

**DOI:** 10.3390/s26041193

**Published:** 2026-02-12

**Authors:** Aristea Athanasopoulou, Nikos Fotiou, Avraam Chatzopoulos

**Affiliations:** 1 Department of Industrial Design and Production Engineering, University of West Attica, 122 41 Athens, Greece; arathanaso@uniwa.gr (A.A.); xatzopoulos@uniwa.gr (A.C.); 2 ExcID Private Company, 113 62 Athens, Greece

**Keywords:** context brokers, LLM, MCP, NGSI-LD API

## Abstract

The rapid proliferation of the Internet of Things (IoT) systems has resulted in large volumes of heterogeneous data that are often difficult to access and exploit due to limited interoperability and complex application programming interfaces. Data spaces address these challenges by providing governed environments for secure and semantically interoperable data sharing, commonly relying on standardized interfaces such as the ETSI NGSI-LD API. While powerful, these interfaces are primarily designed for machine-to-machine interaction and remain difficult to use directly by human operators. In this paper, we propose an architecture that enables natural-language access to IoT data stored in a data space by integrating Large Language Models (LLMs) with the Model Context Protocol (MCP). Experimental results using *fastMCP* and OpenAI API to access a FIWARE-based data space demonstrate that our solution offers accuracy even for prompts that require advanced reasoning.

## 1. Introduction

The proliferation of Internet of Things (IoT) devices has led to the generation of massive volumes of data across domains such as smart cities, manufacturing, energy management, and environmental monitoring. Despite this abundance, the lack of shared semantics and interoperable data-access mechanisms continues to impede large-scale data reuse and value creation. As a result, heterogeneous IoT deployments often remain isolated, limiting the potential for advanced analytics, cross-domain applications, and data-driven innovation.

Data spaces have emerged as a promising response to these interoperability challenges and have already been considered in many applications (see, for example, [1,2,3]). They provide a governed environment in which data can be shared securely, transparently, and under clearly defined usage rules. By enabling structured collaboration among independent organizations, data spaces aim to unlock new forms of data-driven innovation and accelerate digital transformation across sectors [4]. Several international initiatives—including the International Data Spaces Association (IDSA), GAIA-X, and FIWARE—are actively working to establish common architectures, governance models, and interoperability standards. These efforts emphasize not only technical compatibility but also data sovereignty, ensuring that data providers maintain control over how their data is accessed and used.

Access to a data space is often enabled through the ETSI NGSI-LD API [5], a standardized HTTP interface for creating, querying, and updating contextual information. While NGSI-LD provides powerful capabilities for machine-to-machine interactions, it remains difficult for human users to operate directly due to its verbose syntax and the complexity of assembling multi-step queries or workflows. Graphical user interfaces can simplify basic interactions, but they offer limited support for tasks requiring iterative reasoning, dynamic decision-making, or advanced query composition. To unlock the full potential of data spaces for end users, more intuitive and flexible interaction mechanisms are needed.

Large Language Models (LLMs) [6] offer a compelling solution by enabling natural-language interaction with complex systems. Their strengths include the ability to accept unstructured natural-language prompts and generate coherent responses, to reason about intermediate and final outputs, and to interact with users in a dialogue-like manner that incrementally refines requirements and improves the correctness of the produced results. Recent advances have demonstrated their ability to perform sophisticated reasoning with application in the IoT (see, for example, [7,8]). However, traditional LLMs operate as closed systems: they cannot access private data sources or execute actions unless explicitly integrated with external services.

Early attempts to bridge this gap relied on custom *agent-based frameworks*, in which agents are capable of invoking external tools while using LLMs to determine which tool to call and how to parameterize each invocation. A well-known example is HuggingGPT [9], which uses ChatGPT to select and invoke appropriate models from the Hugging Face model repository (https://huggingface.co, accessed on 10 February 2026). Although effective in specific scenarios, such bespoke solutions are difficult to generalize, lack interoperability, and require significant engineering effort to develop and maintain. To illustrate the inefficiencies and limitations of this approach, we introduce a straw man solution for interacting with a data space using LLMs.

A straw man approach

In this approach, users manually describe the structure and interfaces of a data space to an LLM and ask it to generate API calls or even complete applications that can be executed externally to perform the desired task. This method suffers from several important drawbacks:

- It still adds some complexity to the user, who must manually execute generated API calls or integrate generated code with existing systems.
- It introduces security risks, as the LLM may generate API calls that unintentionally modify or delete data within the data space.
- The LLM must be provided with complete and up-to-date knowledge of the data space structure, as it cannot dynamically discover available entities, types, or capabilities.
- The LLM does not directly observe the results of executed API calls, preventing it from performing post-processing, validation, or iterative reasoning based on returned data.

To address these limitations, the *Model Context Protocol* (MCP) [10] introduces a standardized and extensible approach that enables LLMs to interact with external services and data sources in a uniform manner. By defining a clear protocol for capability discovery, tool invocation, and data exchange, MCP provides the missing layer that allows LLMs to function as intelligent interfaces to complex digital ecosystems.

In this work, we explore how MCP can be combined with NGSI-LD to enable natural-language interaction with data spaces, allowing LLMs to autonomously query structured, IoT-generated data. This integration has the potential to significantly enhance the usability and accessibility of data spaces, bridging the gap between human users and the rich semantic data made available through NGSI-LD. The main contributions of this paper can be summarized as follows:

- We develop a solution that enables access to IoT data and supports reasoning over such data using natural language, by leveraging Large Language Models.
- We adopt an extensible and reusable framework, namely the Model Context Protocol (MCP), to enable LLMs to access IoT data, thereby reducing potential errors and limiting the attack surface.
- We design and implement an MCP server capable of reading data from a data space using the ETSI NGSI-LD API, enabling interoperable and extensible access to contextual IoT data.
- We develop an access-control solution that enables per-user, fine-grained authorization and enforces authorization at the MCP server, mitigating security risks arising from malicious or misconfigured LLM behavior.
- We implement and validate the proposed solution using a FIWARE Context Broker, FastMCP, and LLM models by OpenAI.

The remainder of this paper is organized as follows. Section 2 introduces the Model Context Protocol and the concept of data spaces. Related work is discussed in Section 3. Section 4 presents the design of the proposed solution. Section 5 describes the implementation and evaluates the system. Section 6 concludes the paper and outlines directions for future work.

## 2. Background

### 2.1. The Model Context Protocol (MCP)

**Figure 1:**
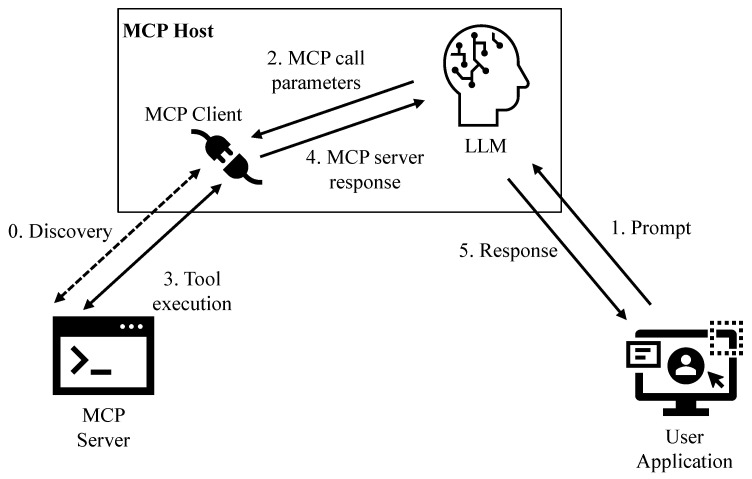
The Model Context Protocol.

MCP follows a client–server architecture in which an *MCP host*—typically an LLM-enabled application such as an intelligent assistant or code-generation platform—connects to one or more *MCP servers* through an *MCP client* (see also Figure 1). Each server exposes a set of primitives that the language model can leverage to interact with external systems. The protocol aims to provide a standardized, extensible, and transparent mechanism enabling LLMs to operate beyond static text generation and engage with real-world data and operations. The most important primitive defined by the MCP specifications is *tools*. Tools represent executable operations that the AI application can invoke on behalf of the language model. Examples include querying a database, processing a file, interacting with a Web service, controlling an IoT actuator, or executing a specific application. Tools enable the LLM to request external actions through explicit, structured invocations, avoiding the need to generate free-form code or natural-language instructions that would otherwise require additional parsing or interpretation by external systems. An MCP server may also provide *resources* and *prompts*. Resources provide access to descriptive or contextual information made available by an MCP server and do not expose executable functionality. Prompts consist of predefined interaction templates or guidance that the LLM can use to structure its communication with users, enabling more consistent and reliable responses.

When the MCP host establishes a connection to a server, it begins with a *discovery phase*. During this phase, the server advertises its available tools, resources, and prompts through structured schema definitions, typically using JSON-based descriptors. Each primitive includes:

- a unique name;
- a natural-language description explaining its purpose and expected use;
- input and output parameters with a natural-language description.

Natural-language descriptions are primarily used to guide the language model’s internal reasoning. Because the model receives a natural-language explanation of each primitive, it can infer which capability is appropriate for a given instruction and how to format the request. The structured parameter definitions ensure that tool invocations are generated with syntactic precision, reducing the potential for execution errors.

After discovery, the MCP host constructs a unified capability registry that combines primitives from all connected servers. From this point on, the model is aware of the set of available actions and contextual data it may use during a conversation.

Once the discovery phase is complete, the integration between the user, LLM, and MCP servers follows a consistent operational pattern:

1. **User Prompt:** The user provides an instruction in natural language, for example, “Retrieve the latest temperature readings from all sensors in Floor 3.”
2. **Model Reasoning and Tool Selection:** The language model analyzes the prompt and determines whether fulfilling the request requires external information or action. Based on the descriptions received during discovery, it identifies the appropriate tool.
3. **Tool Call Construction:** The model generates a structured tool invocation, including the name of the tool and the required input parameters. This call is formatted according to the MCP protocol, ensuring machine-readable interpretation.
4. **Execution:** The MCP client intercepts the tool call and routes it to the corresponding server. The server executes the operation—such as performing a query, running a calculation, or interacting with hardware—and returns a structured response.
5. **Model Integration and Response:** The result is inserted back into the conversation context for the LLM, which may summarize the data, visualize it, or use it to perform subsequent reasoning.

This architecture enables LLMs to operate as interactive agents capable of performing tasks beyond language generation.

### 2.2. Data Spaces and NGSI-LD API

Data spaces provide a structured environment in which multiple stakeholders can share, exchange, and govern data in a controlled and interoperable manner. At the core of a data space is the notion of a *digital entity*, which serves as a virtual representation of a physical or conceptual object. Each entity is identified by a globally unique URI, is associated with a specific *type*, and contains a collection of *attributes*. Attributes may express inherent characteristics of the entity (properties) or links to other entities (relationships). This representation model enables data to be organized in a semantically coherent way, supporting interoperability across heterogeneous systems. A single type may describe a large class of real-world objects, and individual objects may maintain multiple attributes, providing a flexible foundation for large-scale, cross-domain data integration.

**Figure 2:**
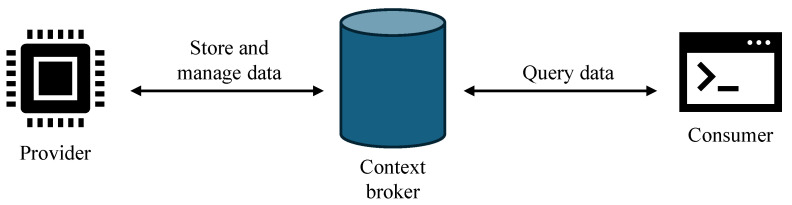
Data space entities.

A data space typically consists of components designed to ensure secure, accountable, and scalable data exchange (see Figure 2). A central element is the *context broker*, which maintains the current values of all entities and exposes interfaces for querying, updating, and subscribing to changes. Data is supplied and managed by a *provider*. Stakeholders who retrieve or process data from the context broker are referred to as *data consumers*. Consumers may request information about individual entities or query collections of entities matching certain criteria, such as type, geographic location, or attribute values.

Interoperable data management within a data space is enabled by the *Next-Generation Service Interfaces–Linked Data* (NGSI-LD) specification developed by ETSI [5]. NGSI-LD defines a RESTful interface for managing and interacting with digital entities using HTTP. In addition to basic CRUD (Create, Read, Update, Delete) functionality, NGSI-LD supports advanced operations such as filtered queries over entity collections, temporal queries for historical data, and subscription management for receiving notifications when specific conditions are met.

## 3. Related Work

Ningyuan et al. [11] leverage MCP to enable an LLM to retrieve IoT measurements. Their architecture introduces multiple MCP servers and an additional “connection server” that mediates communication between the LLM and IoT devices through a custom API layer. Similarly, the solution presented in [12] integrates MCP with a custom API, allowing an LLM to interact with air-quality sensor measurements. In contrast, our approach relies on a single MCP server that exposes multiple tools and—more importantly—interacts with the data space through a standardized API. This removes the need for intermediary services and simplifies integration.

SensorMCP [13] applies MCP to allow LLMs to generate custom tools for specific sensor-processing tasks and subsequently use those tools to complete object-recognition operations in video streams. While effective for its target domain, its scope is limited to vision-based tasks. By incorporating the NGSI-LD API and the broader abstraction of data spaces, our solution enables more general and semantically rich applications extending beyond a single sensing modality.

LLMind [14] allows users to control IoT devices through natural-language prompts, with the LLM generating scripts that are then executed to interact with physical devices. TaskSense [15] and Javis [16] adopt a similar strategy. However, these approaches do not rely on a generic interoperability framework such as MCP. As a result, they require custom integrations and lack the extensibility that MCP-based architectures provide. Our solution, by contrast, builds on a standardized protocol for tool invocation, making it more adaptable to heterogeneous systems.

Retrieval-Augmented Generation (RAG) [17] is a technique that extends the effective context of Large Language Models by augmenting prompts with externally retrieved information. RAG-based approaches have also been explored in the context of LLM interaction with IoT systems [18], where retrieved knowledge is used to enhance the model’s ability to guide users in selecting and configuring appropriate IoT devices for specific tasks. In contrast to our approach, which focuses on enabling LLMs to directly access and reason over live data through structured tool invocation, RAG primarily enriches the model’s contextual knowledge. As such, RAG is complementary to our solution: it can be combined with MCP-based data access to improve the LLM’s reasoning capabilities, for example, by supplying background knowledge, documentation, or domain rules, while relying on MCP tools to obtain up-to-date measurements and execute controlled operations.

TARGE [19] leverages LLMs to generate rules for interacting with trigger–action platforms. The system is designed to integrate with existing trigger–action services, which typically do not provide machine-readable documentation suitable for LLM-based interaction and do not support standardized protocols such as MCP. As a result, TARGE incorporates classification components and model-training pipelines to fine-tune LLMs so that they can generate valid trigger–action rules from natural-language user prompts. TARGE highlights the challenges of interacting with real-world systems in the absence of standardized interaction models and well-defined interfaces for LLMs. In contrast, our approach relies on MCP to expose system capabilities as structured tools with explicit semantics. This enables LLMs to invoke APIs in a controlled and predictable manner without requiring task-specific fine-tuning or additional model training. On the other hand, our approach assumes the availability—or the development—of an MCP server that bridges LLMs with existing services. While this introduces an integration effort, it shifts complexity away from model training and toward standardized, reusable interfaces that improve interoperability and long-term maintainability.

Our solution focuses on enabling secure and interoperable interaction with a data space and presenting query results back to the user through natural-language interfaces. While our work does not address large-scale data collection or stream summarization, it can be leveraged as a foundational component by systems that operate at those layers. For example, Flash-Fusion [20] provides a solution that translates a user prompt into a data collection strategy, collects and pre-processes relevant IoT data, and feeds the resulting summaries to an LLM in order to generate a final response. In its current form, Flash-Fusion does not rely on a standardized interaction model or data representation format, such as MCP and NGSI-LD, nor does it explicitly address access control and authorization concerns. Our solution is complementary and could be integrated into such systems to provide a standardized, secure, and semantically grounded mechanism for accessing IoT data spaces. In this combined setting, Flash-Fusion could focus on optimizing data acquisition, aggregation, and latency, while our MCP-based approach would handle authenticated and authorized access to data spaces, structured data retrieval, and interoperability across heterogeneous IoT platforms.

Other systems focus on improving an LLM’s ability to generate correct API calls. Gorilla [21] and ToolLLM [22] fine-tune language models specifically to produce accurate API invocations from natural-language instructions. These approaches are complementary to ours: an LLM optimized for structured API calling could enhance the reliability of MCP tool invocation. Similarly, EASYTOOL [23], which generates concise and informative tool descriptions to improve tool selection, can further strengthen our architecture. Together, these complementary techniques can enhance both the accuracy and usability of LLM–tool interactions within our MCP-based design.

## 4. Design

### 4.1. Reference Data Space

In this work, we consider a data space consistent with the description provided in Section 2.2 and instantiated in the context of a smart city use case. In this setting, heterogeneous IoT devices deployed across the urban environment continuously collect measurements and publish them to the data space. The data space is administered by a trusted authority, such as the city hall, which is responsible for operating the context broker. Each IoT device is associated with a specific physical location, such as a public building, a road segment, or an urban area, and this spatial information is explicitly represented within the data space. Access to data is restricted to authenticated users.

For the remainder of this paper, we rely on a reference data space populated with a set of entity types and instances, which are used for design illustration and experimental evaluation. The data space is structured around ten entity types representing IoT device types and one entity type representing buildings. Each type comprises two objects, each uniquely identified by a URL-encoded object identifier and associated with a set of attributes relevant to its function. All entity types corresponding to IoT devices include a relationship attribute named located_in. The value of this attribute is the identifier of the building in which the device is located.

### 4.2. Architecture

**Figure 3:**
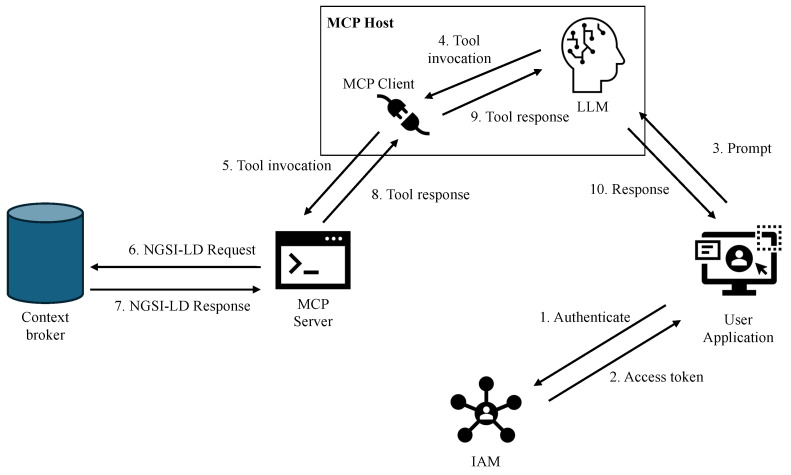
High-level overview of the proposed architecture.

Figure 3 provides a high-level overview of the proposed architecture. In our approach, users access the data space through an MCP host equipped with a Large Language Model (LLM). The architecture includes a user-facing application that provides an interface through which users can submit requests in natural language. This application communicates with the LLM hosted by the MCP environment, which processes user requests and generates responses grounded in data retrieved from the data space via structured MCP tool invocations.

The MCP host integrates an LLM that is augmented with an MCP client. This client establishes a connection to an MCP server, which acts as an intermediary between the LLM and the data space. The MCP server exposes an HTTP-based API and provides tools that encapsulate the operations required to interact with the underlying context broker. Internally, the MCP server translates tool invocations into NGSI-LD API calls, allowing it to retrieve data stored in the data space. The specific tools exposed by the MCP server are described in detail in Section 4.3.

User identity and access control are managed by a dedicated Identity and Access Management (IAM) system. Users authenticate through this system and obtain an access token, which is subsequently used during interactions with the MCP host system. This token serves a dual purpose: it enables user authentication and conveys fine-grained authorization information that determines which data and operations the user is allowed to access. Security-related mechanisms, including authentication, authorization, and policy enforcement, are discussed in Section 4.6.

The interaction flow proceeds as follows (see also Figure 3). First, a user authenticates with the user management system (step 1) and receives an access token (step 2). The user then submits a natural-language prompt through the user application interface, which is transmitted, together with the access token, to the MCP host (step 3). The LLM analyzes the prompt and, when external data access is required, selects one or more appropriate MCP tools based on the capabilities advertised by the MCP server (step 4). The MCP client forwards the corresponding tool invocation, together with the user-provided access token, to the MCP server (step 5). The MCP server validates the request, enforces authorization constraints, and executes the required NGSI-LD operations against the context broker (step 6). The results (step 7) are returned to the MCP client (step 8) and injected into the LLM’s context (step 9), enabling the model to generate a final response for the user (step 10). Steps 4–9 may be repeated more than once in the context of a single prompt.

### 4.3. MCP Tools

#### 4.3.1. The read()

Tool The MCP server in our architecture exposes a tool named read(), which provides a unified interface for retrieving data from the context broker through the NGSI-LD API. The tool supports both object-specific and type-based queries and abstracts the complexity of constructing NGSI-LD requests from the LLM.

The read() tool retrieves one or more NGSI-LD entities depending on the parameters provided. Its input parameters are defined as follows:

- **Type identifier (optional):** A string representing the NGSI-LD type identifier used to retrieve all entities of a given type (e.g., “plug”).
- **Object identifier (optional):** A string representing the URI of a specific entity to be retrieved (e.g., “urn:plug1”).
- **Attributes (optional):** A list of attribute names specifying which attributes should be included in the response. If omitted, all available attributes are returned.
- **Filters (optional):** A list of filter expressions used to restrict the result set based on attribute values. Each filter is expressed as a string of the form <attribute operator value>, for example, temperature>30. Supported operators include equality and inequality comparisons (==, !=, <, <=, >, >=).

At least one of type_id or object_id must be specified. The tool returns a JSON array containing one or more entities represented according to the NGSI-LD data model.

Table 1 presents example natural-language prompts and the corresponding structured invocations of the read() tool.

#### 4.3.2. The get_types() Tool

The get_types() tool retrieves a filtered list of entity types available in the data space. For each type, the tool returns the set of attributes associated with entities of that type, along with a human-readable description. This information allows the LLM to understand the structure and semantics of the data exposed by the data space.

The get_types() tool accepts as input a list of keywords, which are used as search criteria to identify entity types relevant to a given user prompt. By restricting the returned results to types that match the provided keywords, the tool reduces the amount of information included in the LLM context. This is important for preserving the available context window, i.e., the maximum amount of text (measured in tokens) that an LLM can process as input in a single invocation.

To support keyword-based type discovery, we introduce a data-space metadata file that describes all available entity types together with their corresponding attributes and textual descriptions. In our current implementation, this metadata file is created manually. However, given that NGSI-LD relies on JSON-LD and explicitly encodes semantic relationships, the construction of such a metadata representation can be automated. The automated generation of this file is left as future work.

### 4.4. Prompt and Tools Descriptions

A key component of an MCP server is the prompt that describes how the MCP host should use the capabilities exposed by the server, together with the descriptions of the available tools and their corresponding parameters. These descriptions play a crucial role in enabling the LLM to correctly interpret user requests and map them to appropriate tool invocations. A complete specification of the prompts and tool descriptions used in our implementation is provided in Appendix A.

The prompt supplies explicit instructions to the MCP host on how to reason about user requests and how to combine multiple tool invocations when necessary, without requiring additional user intervention. For instance, our prompt includes the following instruction: *If the user asks you for an attribute or measurement and you do not know to which type it belongs, use get_types to find out.* As a result, when a user submits a request such as *What is the temperature in building2?*, the MCP host first invokes the get_types() tool to identify which entity type provides a temperature attribute. Upon determining that temperature measurements are associated with entities of type thermometer, the MCP host subsequently invokes the read() tool with the parameters type_id=thermometer and filters=["located_in==building2"] to retrieve the relevant data.

### 4.5. Reasoning

A key capability of LLMs is their ability to perform reasoning by combining information retrieved from external sources with knowledge implicitly encoded during training. In our architecture, reasoning does not rely on explicitly scripted decision rules embedded in the prompt. Instead, the LLM infers how to interpret user intent, which data to retrieve, and how to process the retrieved values in order to produce a meaningful response.

One important aspect of this behavior is the LLM’s ability to leverage background knowledge that is not explicitly represented in the data space. For example, in our testing dataset, building entities include only a postal code and an address but do not explicitly store the name of the city. Nevertheless, users can issue queries referring to cities (e.g., asking about buildings in a specific city), and the LLM is able to infer the city associated with a given postal code based on knowledge acquired during training. This enables queries that combine retrieved data with external knowledge without requiring additional enrichment of the data space.

The LLM is also capable of performing operations over retrieved results. For instance, users can request aggregated values, such as the average temperature of a building or the sum of measurements across a set of devices. In such cases, the LLM retrieves the relevant measurements through MCP tool invocations and then applies the required aggregation operation to produce the final result. Similarly, when asked to identify the hottest building, the LLM infers that it should retrieve temperature measurements for multiple buildings and determine the building associated with the highest value. When asked to identify a building with a potential water leakage, the LLM deduces that this corresponds to unusually high water consumption and retrieves and compares the relevant measurements accordingly.

Finally, reasoning often requires multiple, coordinated interactions with the MCP server. The LLM may need to discover relevant entity types, retrieve measurements from different devices, and combine or compare results across several tool invocations before producing a response. This ability to plan and execute multi-step data access and post-processing workflows illustrates how LLMs can move beyond simple data retrieval and provide higher-level, semantically rich answers grounded in the data space, without relying on rigid, hand-crafted rules.

### 4.6. Security

User applications interacting with the proposed system provide an *access token* to the LLM, which is subsequently used to access the MCP server. Many existing solutions rely on long-lived bearer tokens, where possession of the token alone is sufficient to prove authorization. This approach introduces significant security risks in MCP-based architectures, as access tokens may be exposed to the LLM during processing and could be leaked, reused, or misused by unauthorized parties.

To mitigate these risks, our architecture adopts short-lived access token proofs of possession that are cryptographically bound to a public key controlled by the user application. This mechanism is based on OAuth 2.0 Demonstrating Proof of Possession (DPoP) [24], which ensures that an access token can only be used by an entity that proves possession of the corresponding private key.

#### 4.6.1. DPoP-Based Authentication Flow

The security workflow proceeds as follows. A user first authenticates with the Identity and Access Management (IAM) system and obtains an OAuth 2.0 access token. This access token encodes the user’s authorization claims and embeds a public key $K_{\mathrm{pub}}$ associated with the user application. The corresponding private key $K_{\mathrm{priv}}$ is securely stored by the user application and never shared.

Whenever the user application initiates an interaction that may trigger access to the MCP server, it generates a *proof of possession*. In accordance with [24], this proof is a JSON Web Token (JWT) digitally signed using $K_{\mathrm{priv}}$. The JWT includes, among others, the following claims:

- an *audience* (aud) claim set to the URL of the MCP server,;
- a timestamp and expiration time defining a short validity window;
- a unique identifier to prevent replay attacks.

Formally, the proof can be expressed as

$$\mathrm{DPoP}={\mathrm{Sign}}_{K_{\mathrm{priv}}}\left(\mathrm{JWT}\left(\mathrm{aud},\mathrm{iat},\exp,\ldots \right)\right)$$

The user application includes both the access token and the generated DPoP proof in each request sent to the MCP host.

#### 4.6.2. Verification at the MCP Server

An MCP client forwards the access token and the accompanying DPoP proof to the MCP server. The server then performs a sequence of validation steps:

- It verifies that the access token is syntactically valid and issued by a trusted IAM.
- It checks that the authorization claims contained in the access token permit the requested operation.
- It validates that the DPoP proof is within its declared validity period.
- It verifies the signature of the DPoP proof using the public key $K_{\mathrm{pub}}$ embedded in the access token, ensuring that the requester possesses the corresponding private key.
- It confirms that the aud claim of the DPoP proof matches the MCP server’s URL.

Only if all checks succeed is the request authorized and forwarded for execution.

By binding access tokens to a cryptographic key pair and requiring a fresh proof of possession for each request, the proposed approach significantly reduces the risk of token theft and replay. Even if an access token is exposed to the LLM or intercepted by an attacker, it cannot be reused without access to the corresponding private key. This design is particularly well-suited for LLM-based systems, where minimizing the impact of credential leakage is essential.

### 4.7. Mitigating Hallucinations

During the implementation and evaluation of the proposed system, we observed that, on rare occasions, the LLM attempted to invoke MCP tools using non-existing entity types, attributes that are not defined for a given type, or filtering expressions that rely on unsupported operators. Such behavior is a well-known manifestation of LLM hallucinations, particularly when models attempt to generalize beyond the explicitly exposed capabilities of the system.

In our architecture, these hallucinations are not harmful from a safety perspective, as the MCP server only exposes read-only operations. Consequently, no modification of the data space can occur as a result of an erroneous tool invocation. In the absence of additional safeguards, such hallucinations would typically result either in error messages generated by the MCP server or in empty responses returned by the data space, both of which can degrade the user experience and confuse the LLM during subsequent reasoning steps.

To address this issue, and based on extensive trial-and-error experimentation—especially with older or less capable models—we introduced a set of safeguard mechanisms at the MCP server. These safeguards detect common hallucination patterns and provide explicit feedback to the LLM. Specifically, when the LLM requests a non-existing entity type or an attribute that is not defined for a given type, the MCP server returns a clear error message indicating that the requested type or attribute does not exist, instead of forwarding the request to the data space and returning an empty result. This explicit feedback allows the LLM to revise its assumptions and issue a corrected tool invocation.

Similarly, when the LLM provides an object identifier that is not properly URL-encoded or specifies a filtering operator that is not supported or invokes a tool with the wrong number of parameters, the MCP server responds with a descriptive error message that includes guidelines on the expected input. In addition to validation and error reporting, the MCP server performs limited post-processing of filter expressions, such as automatically inserting quotation marks where required and applying URL-escaping to parameter values when appropriate.

For example, in earlier and less capable models, the LLM occasionally made incorrect assumptions about how the read() tool should be invoked. A common example was the simultaneous inclusion of both a type identifier and an object identifier in a single invocation, even though the tool description specifies that either a type identifier or an object identifier should be provided. While NGSI-LD itself allows requests that include both parameters—and would typically return an empty result set if the type does not exist—our MCP server enforces stricter validation. When the LLM provided a non-existing type together with an object identifier, the MCP server returned an explicit error message indicating that the specified type does not exist, instead of forwarding the request to the data space and returning an empty response. This explicit feedback enabled the LLM to correct its behavior in subsequent invocations.

We also observed hallucinations in more recent models, particularly when conversational context was preserved across multiple requests. In such cases, the LLM tended to infer associations between attributes and entity types based on prior interactions. For example, after learning that a type named “thermometer” exists, the model would sometimes assume that any request involving the attribute “temperature” should be issued with type_id=“thermometer”. To validate that this behavior, we intentionally modified the data space during testing by assigning the attribute “temperature” to a different type (e.g., a plug). Under these conditions, the LLM generated an incorrect tool invocation, requesting the “temperature” attribute from the “thermometer” type. The MCP server detected that the requested attribute was not defined for that type and returned a clear error message stating that the type “thermometer” does not include the “temperature” attribute. In response, the LLM invoked the get_types() tool to discover which entity type actually exposes the requested attribute and then issued a corrected read() invocation.

## 5. Implementation and Evaluation

A proof of concept of the proposed solution has been implemented using the FIWARE Orion Context Broker (https://github.com/FIWARE/context.Orion-LD, accessed on 10 February 2026) as the data-space backend. The MCP server was developed using the FastMCP framework (https://gofastmcp.com, accessed on 10 February 2026). For the Large Language Model component, we relied on the OpenAI platform (https://openai.com, accessed on 10 February 2026) and employed various models.

### 5.1. Proof-of-Concept Implementation

We implemented a proof of concept that allows users to interact with the proposed system through a standard web browser. The system provides a chat-like user experience in which users can submit natural-language prompts and receive responses derived from the underlying data space. The front-end consists of a web-based user interface that displays the dialogue and forwards user input to a backend service. The backend is implemented using FastAPI (https://fastapi.tiangolo.com/, accessed on 10 February 2026) and is responsible for coordinating interactions between the user interface, the Large Language Model, and the remaining system components, as well as for securely storing access tokens.

The backend communicates with the OpenAI platform to invoke the LLM and is configured with the necessary API credentials during an initial setup phase. The same setup phase includes configuring the Identity and Access Management system, which in our implementation is based on the Keycloak open-source platform (https://www.keycloak.org/, accessed on 10 February 2026), with the required user accounts and authorization settings. The MCP server is deployed separately and connected to a FIWARE Context Broker.

The user interaction flow proceeds as follows. A user first authenticates with the Identity and Access Management system, and upon successful authentication, an access token is issued and stored by the backend component. The user then accesses the web-based interface and submits a natural-language prompt. This prompt is received by the backend, which forwards it to the OpenAI platform using the *responses* API (https://platform.openai.com/docs/api-reference/responses, accessed on 10 February 2026). Along with the user prompt, the backend provides the LLM with the MCP tool configuration, including the URL of the MCP server and a token corresponding to a freshly generated DPoP proof.

Based on the prompt and the provided tool configuration, the LLM may decide to invoke one or more MCP tools. In such cases, the MCP server is contacted directly using the supplied credentials, and the requested operations are executed against the FIWARE Context Broker. The results of these operations are returned to the LLM, which incorporates them into its reasoning process and generates a final response. This response is sent back to the backend and subsequently displayed to the user through the web interface. This proof-of-concept implementation demonstrates the feasibility of end-to-end, secure, and interactive access to a data space using LLMs and the Model Context Protocol.

In the current proof-of-concept implementation, the MCP server performs a basic validation of the security credentials by verifying that the access token associated with the DPoP proof has been issued by a trusted Identity and Access Management server. However, more advanced authorization mechanisms can be used (see, for example, [25]). In our deployment, a clear separation of responsibilities is enforced between the client-side and server-side components of the user application. The web-based user interface, accessed through a standard browser, is limited to presenting the chat interaction and forwarding user inputs to the backend service. It does not store, generate, or process any security-sensitive material. All cryptographic credentials, including access tokens and the private key used for generating the DPoP proof, are managed exclusively by the backend component, which operates in a secure environment. The backend is responsible for generating DPoP proofs and attaching them to requests sent to the MCP server on behalf of authenticated users.

### 5.2. Performance Evaluation

#### 5.2.1. Evaluation Setup

To evaluate the performance of the proposed solution (Evaluation results can be replicated using the code available at https://github.com/iot-data-space/mcp, accessed on 10 February 2026), we constructed a synthetic data space and a controlled set of test prompts. The data space is composed of building entities and ten distinct types of IoT devices. Our data space includes 100 devices, and on average, each building is associated with 20 devices. Each building entity includes an address consisting of a street name and number, as well as a postal code, but intentionally omits the city name. This design choice allows us to test the LLM’s ability to combine retrieved data with external knowledge learned during training.

For the evaluation, we prepared a list of 30 prompts and implemented a tool to automate interactions with the LLM. The prompts are grouped into six categories reflecting increasing levels of complexity. The first category (c1) includes prompts requesting the value of a specific measurement from an individual device, such as requesting the consumption reading of a particular smart plug. The second category (c2) consists of prompts that require calculating the average value of a given measurement for all devices located in a specific building. The third category (c3) includes prompts that ask for the sum of a measurement across all devices of a given type. The fourth category (c4) mirrors the third but deliberately introduces misspellings in the device type names in order to assess the robustness of the LLM in handling imperfect input. The fifth category (c5) contains prompts that require conditional aggregation, such as computing sums or averages of measurements for devices located in buildings that satisfy additional constraints (e.g., buildings containing other devices with specific properties). Finally, the sixth category (c6) includes prompts that require reasoning or the use of external knowledge, such as identifying the Greek city associated with the largest number of buildings.

All prompts are formulated so that the expected response is a single number or a single word. This constraint enables fully automated validation of the LLM outputs. The correct answers for all prompts were computed manually and are used as a reference during evaluation. The full list of prompts is provided in the appendix. Our evaluation script interacts with multiple LLM configurations, including gpt-5.2, gpt-5-mini, gpt-5-nano, and the older gpt-4.1. Each model has different capabilities and different costs associated with it. All prompts are executed using a separate, clean context.

In addition, we implemented the straw man approach described in the introduction to provide a baseline for comparison. In this setup, three LLMs—ChatGPT using gpt-5.2, Google Gemini 3, and Claude using Sonnet 3—were instructed to generate a Python 3.9 script that directly interacts with the data space in order to retrieve the information needed to answer the prompts. The prompt provided to each model included both the list of evaluation prompts and the data space metadata file described in Section 4.3.2. The generated scripts are referred to in the results as S1, S2, and S3, respectively.

#### 5.2.2. Accuracy

Table 2 reports the number of correct responses as a ratio of the total number of prompts for each prompt category. Accuracy is measured using an exact-match comparison between the model output and the manually computed reference answer. The columns labeled S1, S2, and S3 correspond to the three implementations of the straw man approach described earlier.

Overall, the results indicate that recent LLMs achieve high accuracy across most prompt categories. For categories c1 to c5, which involve direct retrieval, aggregation, and conditional filtering of measurements, all recent models achieve near-perfect or perfect accuracy, even when using the straw man approach. This suggests that, for relatively well-structured tasks, modern LLMs are capable of producing correct results. More challenging behavior is observed in category c6, which includes prompts requiring reasoning or the use of external knowledge. In this category, accuracy drops across all approaches, with larger and more capable models outperforming smaller ones. Notably, the older gpt-4.1 model fails in approximately half of the prompts across multiple categories, including complete failure in c5 and c6. This highlights the substantial progress achieved in more recent LLM models in terms of robustness, reasoning capabilities, and tolerance to imperfect input.

#### 5.2.3. Cost

The cost of executing LLM-based queries is primarily determined by the number of *input tokens* and *output tokens* processed by the model. Input tokens correspond to all textual information provided to the model in a single invocation, including the user prompt, system instructions, tool descriptions, and any contextual information carried over from previous interactions. Output tokens correspond to the text generated by the model as a response, including intermediate reasoning steps and the final answer returned to the user. Most commercial LLM platforms charge separately for input and output tokens, typically on a per-million-token basis.

Table 3 summarizes the cost of input and output tokens for the gpt-5 family models used in our evaluation. The prices shown are expressed in U.S. dollars per one million tokens and correspond to the pricing as of January 2025.

**Figure 4:**
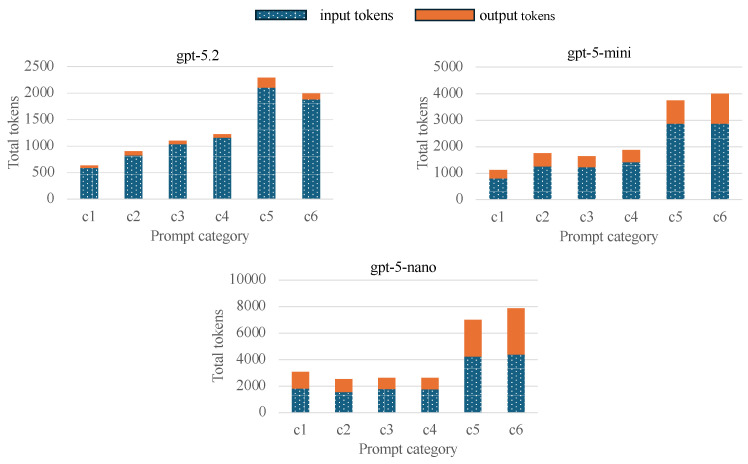
Token consumption per model.

Figure 4 presents the token consumption per model. Although all models received identical prompts and produced identical outputs, we observed significant differences in token consumption. Smaller models consistently consumed more tokens than larger ones. This behavior can be attributed to differences in reasoning efficiency: smaller models require more explicit contextual information, perform additional intermediate reasoning steps, and more frequently rely on tool discovery and verification. In contrast, larger models are able to infer the appropriate actions with less contextual scaffolding, resulting in lower overall token usage. These findings highlight that token efficiency is not solely determined by prompt length or output size, but also by model capacity and reasoning efficiency.

#### 5.2.4. Delay and MCP Tool Invocations

To better understand the performance characteristics of the proposed solution, we analyze both the end-to-end execution delay and the number of MCP tool invocations required to answer user prompts. Table 4 reports the average execution time per prompt category, while Table 5 reports the average number of MCP tool invocations issued by the LLM for the same categories.

The results show a clear correlation between execution delay and the number of MCP tool invocations. Prompt categories that require simple data retrieval (c1) are typically resolved with a single tool invocation for the larger models, resulting in lower response times. In contrast, smaller models invoke MCP tools more frequently for the same tasks, which substantially increases overall delay. This effect is particularly pronounced for gpt-5-nano, which invokes, on average, three tools even for the simplest prompt category and exhibits more than three times the latency of gpt-5.2 in c1.

As the complexity of the prompts increases, both the number of tool invocations and the execution delay increase across all models. Categories c5 and c6, which involve conditional aggregation and reasoning, exhibit the highest delays and the largest number of tool calls. These categories often require the LLM to discover relevant entity types, retrieve multiple measurements, and apply reasoning rules, resulting in multiple sequential interactions with the MCP server. Again, smaller models exhibit a significantly higher number of tool invocations, which directly translates into longer response times.

Overall, these results indicate that execution delay is dominated not only by the cost of individual MCP tool calls but also by the efficiency with which an LLM plans and minimizes such calls. Larger models are able to infer the appropriate data access strategy with fewer interactions, leading to lower latency. In contrast, smaller models compensate for their reduced reasoning capabilities by issuing additional tool invocations, increasing both execution time and system load.

### 5.3. The Impact of Hallucinations

As discussed in Section 4.7, the MCP server incorporates extensive tool descriptions, validation logic, and descriptive error messages designed to mitigate LLM hallucinations. To quantitatively assess the impact of hallucinations and to demonstrate the importance of proper tool descriptions and instructions, we conducted an additional experiment in which these safeguards were intentionally weakened. Specifically, we created a modified version of the MCP server that exposes only the raw documentation of the available tools, without providing higher-level usage instructions. In this configuration, the LLM still has access to the tool signatures but lacks guidance on how tools should be combined, which parameters are mutually exclusive, and how to recover from invalid invocations.

Table 6 reports the number of correct responses per prompt category when using gpt-5.2 with this configuration. Despite being a recent and capable model, gpt-5.2 exhibits a noticeable increase in hallucinations under these conditions. In particular, the model more frequently invokes tools using non-existing entity types, attributes that are not defined in the data model, or incorrect combinations of parameters. These hallucinations directly reduce the overall accuracy compared to the results obtained with the full prompt and validation setup. In addition to reduced correctness, we observed a significant increase in the number of MCP tool invocations. Even when the final response was correct, the absence of explicit instructions caused the model to explore the tool space more extensively, issuing redundant or corrective calls. This effect is especially pronounced for reasoning-intensive prompts (category c6), where the model required approximately twice as many MCP tool invocations to converge to a response compared to the fully guided configuration.

### 5.4. Security Evaluation

We evaluate the security of the proposed solution against an attacker who has compromised the MCP host and attempts to use this position to access the MCP server. This threat model is particularly relevant because the MCP host processes user prompts and access tokens.

We assume an adversary A with full control over the MCP host. Concretely, A can: (i) observe and modify any messages sent by the MCP client to the MCP server, (ii) read any data available to the host (including access tokens if they appear at the host), and (iii) initiate arbitrary new requests to the MCP server. However, A does *not* compromise the user application that holds the DPoP private key and thus does not learn the private key $K_{\mathrm{priv}}$. The security goal is to prevent A from successfully invoking MCP tools at the MCP server as the user, unless A can prove possession of the user application’s private key. In other words, exposure of bearer-like credentials at the host should not be sufficient for authorization.

Our solution relies on OAuth 2.0 DPoP [24]. At authentication time, the IAM issues an access token AT that is bound to a public key $K_{\mathrm{pub}}$ controlled by the user application (the corresponding private key $K_{\mathrm{priv}}$ never leaves the user application). In practice, this binding is carried in the token (e.g., via a confirmation claim), enabling the MCP server to extract or verify the associated public key.

For each request to the MCP server, the user application constructs a DPoP proof:

$$P\leftarrow {\mathrm{Sign}}_{K_{\mathrm{priv}}}\left(\mathrm{JWT}(\mathrm{aud},\mathrm{iat},\exp,\mathrm{jti},\ldots )\right),$$

where:

- aud identifies the intended audience (the MCP server URL),
- iat and exp define a short validity window,
- jti is a unique identifier used for replay detection.

The request is accepted only if the MCP server validates AT and verifies *P* using $K_{\mathrm{pub}}$ embedded (or referenced) by AT.

We provide a proof sketch showing that, under standard cryptographic assumptions, compromising the MCP host is insufficient for A to obtain unauthorized access to the MCP server.

We assume:

1. The signature scheme used to sign DPoP proofs is secure.
2. The IAM issues valid access tokens only after successful user authentication and binds each token to the correct $K_{\mathrm{pub}}$.
3. The MCP server enforces DPoP verification and rejects reused proofs based on jti (within an appropriate server-side replay cache window) and rejects expired proofs based on exp.

Let A be an adversary controlling the MCP host but not possessing $K_{\mathrm{priv}}$. Then, A cannot generate a request that is accepted by the MCP server under the victim user’s authorization context beyond replaying an already-valid request within its short validity window.

**Proof.**  To be accepted, a request must include an access token AT and a DPoP proof *P* such that: (i) AT is valid and authorizes the requested operation, (ii) *P* verifies under the $K_{\mathrm{pub}}$ bound to AT, is unexpired, and targets the MCP server via aud.

If A attempts to create a fresh accepted request, it must produce a valid *P* for some message *m* that includes the required claims (at least aud, iat, exp, and jti). Because A does not know $K_{\mathrm{priv}}$, producing such a proof amounts to forging a signature under $K_{\mathrm{pub}}$, contradicting the signature security assumption. Therefore, A cannot produce a new valid proof.

The only remaining strategy is replay: A may copy a previously observed pair (AT,P) from the host and resend it. This is bounded by (a) the short lifetime enforced via exp, and (b) replay detection through jti. Once a proof has been accepted, subsequent uses of the same jti are rejected, and after expiration, the proof is invalid. Hence, even with host compromise, the adversary cannot sustain unauthorized access.    □

#### Implications for the Compromised-Host Scenario

Under the considered threat model, bearer tokens alone would allow A to impersonate the user simply by extracting AT from the host. In contrast, our DPoP-based design ensures that possession of AT is insufficient: the MCP server additionally requires a fresh proof *P* signed with $K_{\mathrm{priv}}$, which the attacker cannot generate if the user application is not compromised. Consequently, an attacker who only controls the MCP host cannot invoke MCP tools on the MCP server as the user, except for narrowly bounded replay attempts mitigated by proof expiration and replay detection.

### 5.5. Discussion

Large Language Models provide an intuitive and flexible interface for interacting with data spaces. By relying on natural language, users can express information needs without detailed knowledge of underlying data models or APIs. This flexibility lowers the barrier to entry for non-expert users and enables more exploratory interactions. Moreover, LLMs exhibit a degree of robustness to imperfect user input. For example, they can often infer the intended meaning of misspelled or ambiguous attribute names, such as interpreting a request for *“temprature”* as a request for the *“temperature”* attribute.

At the same time, the effectiveness and safety of LLM-driven interaction depend heavily on the quality of the instructions and constraints provided to the model. From the perspective of the MCP server and prompt design, precise and explicit guidance is essential. In our implementation, the LLM had to be explicitly instructed not to cache retrieved attribute values and to always obtain fresh measurements from the MCP server, ensuring consistency with the current state of the data space. Without such instructions, the model might reuse outdated values, leading to incorrect or misleading responses.

Similarly, the LLM required domain-specific knowledge to correctly interpret certain attributes and relationships. For instance, the located_in attribute plays a special role in representing spatial relationships between entities and locations. Explicitly informing the LLM of this semantic meaning was necessary to enable correct filtering and reasoning over location-based queries. These examples highlight that, while LLMs offer powerful abstraction and reasoning capabilities, their correct operation in structured environments relies on carefully crafted prompts, well-defined tool descriptions, and explicit semantic conventions.

An important aspect that warrants further consideration is the robustness of LLM-based interfaces against emerging risks such as prompt injection and hallucination. Malicious or carefully crafted user inputs may attempt to manipulate the model into ignoring system-level instructions or invoking tools in unintended ways. Although our architecture mitigates the impact of such attacks by enforcing authorization and access control at the MCP server, prompt injection remains a challenge that must be addressed through careful prompt design, strict separation between user and system instructions, and continuous validation of tool invocations. Despite the fact that our system exposes only read operations, malicious prompts may still have an impact on system stability and availability. For example, we observed that older versions of the FIWARE Context Broker could crash when presented with malformed filter expressions. In such cases, a malicious user could craft a prompt that induces the LLM to generate an invalid filtering condition, potentially leading to denial-of-service behavior. Similarly, a malicious prompt could be designed to trigger unhandled edge cases or implementation bugs in the MCP server library or to exhaust MCP server resources by causing the LLM to issue a large number of tool invocations or repeated requests that systematically result in errors, thereby filling server logs and degrading performance.

Similarly, LLM hallucinations may lead to incorrect interpretations of data or fabricated explanations, particularly when responses rely on inferred knowledge rather than retrieved facts. In this context, constraining the LLM to ground its responses in data obtained through MCP tools and clearly distinguishing between retrieved data and inferred conclusions are essential. Finally, the dynamic nature of LLM behavior motivates the need for continuous monitoring and logging of interactions, both to detect anomalous patterns and to support auditing, debugging, and iterative improvement of prompts and tool descriptions over time.

## 6. Conclusions

In this paper, we leveraged Large Language Models (LLMs) to enable intuitive, secure, and interoperable access to IoT data stored in a data space. By integrating LLMs with the Model Context Protocol, we demonstrated how natural-language interaction can be combined with structured tool invocation to bridge the gap between human users and NGSI-LD–based data spaces. The proposed architecture allows users to query and reason over contextual IoT data without requiring direct knowledge of underlying APIs, data models, or interaction protocols.

We presented the design and implementation of an MCP server that exposes data-space operations as well-defined tools and translates LLM-generated invocations into NGSI-LD requests executed against a FIWARE Context Broker. To address the security challenges inherent in LLM-based systems, we incorporated an Identity and Access Management solution based on OAuth 2.0 Demonstrating Proof of Possession, enabling per-user, fine-grained authorization, and reducing the risks associated with access-token exposure, even in the presence of potentially misconfigured or compromised LLM hosts. A complete proof-of-concept implementation using FastMCP, ChatGPT, and Keycloak demonstrated the feasibility of the approach in an end-to-end setting.

Through an extensive experimental evaluation, we showed that the proposed system supports a wide range of data-access and reasoning tasks with high accuracy across multiple LLM configurations. Our results highlight important trade-offs between accuracy, token consumption, execution delay, and the number of MCP tool invocations. In particular, larger models achieve lower latency and reduced tool usage by planning data-access strategies more efficiently, while smaller models require additional context and interactions to reach correct results. These findings emphasize that cost and performance are influenced not only by prompt size or output length, but also by the reasoning efficiency of the underlying model. Additionally, our results showed that hallucinations, while rare with recent models under well-designed prompts and safeguards, can still emerge when guidance is insufficient; therefore, prompt design and server-side validation are not merely usability enhancements but essential components for achieving both correctness and efficiency in LLM-driven interaction with data spaces.

Finally, we demonstrated that LLMs can perform implicit reasoning by combining retrieved data with background knowledge acquired during training, enabling advanced queries that involve aggregation, comparison, and inference without relying on rigid, hand-crafted rules. Future work will focus on extending the MCP toolset to support more advanced query patterns, temporal analytics, and controlled write operations; investigating large datasets exceeding LLM’s context window; integrating automated generation of data-space metadata; exploring the use of local and hybrid LLM deployments; and investigating agentic applications that can autonomously act upon LLM outputs while remaining within the security and governance constraints of data spaces.

## Figures and Tables

**Table 1:** Example natural-language prompts and corresponding read() tool invocations.

Prompt	Tool Invocation
What are the measurements of urn:thermometer1?	read(object_id="urn:thermometer1")
What is the temperature measured by urn:thermometer1?	read(object_id="urn:thermometer1", attributes=["temperature"])
What is the temperature measured by thermometers in building2?	read(type_id="thermometer", attributes=["temperature"], filters=["located_in==building2"])

**Table 2:** Number of correct responses per prompt category.

	gpt-5.2	gpt-5-mini	gpt-5-nano	gpt-4.1	S1	S2	S3
**c1**	5/5	5/5	5/5	5/5	5/5	5/5	5/5
**c2**	5/5	5/5	5/5	3/5	5/5	5/5	5/5
**c3**	5/5	5/5	5/5	4/5	5/5	5/5	5/5
**c4**	5/5	5/5	5/5	3/5	5/5	5/5	5/5
**c5**	5/5	5/5	5/5	0/5	5/5	4/5	5/5
**c6**	4/5	4/5	2/5	0/5	1/5	1/5	3/5

**Table 3:** Cost of input and output tokens per model (USD per 1 M tokens, January 2025).

	Input Tokens ($/1 M)	Output Tokens ($/1 M)
gpt-5.2	1.75	14.0
gpt-5-mini	0.25	2.0
gpt-5-nano	0.05	0.4

**Table 4:** Average execution time in seconds per prompt category.

Category	gpt-5.2	gpt-5-mini	gpt-5-nano
**c1**	6.22	9.76	22.22
**c2**	7.79	16.73	19.18
**c3**	7.37	13.09	16.37
**c4**	7.47	13.34	15.72
**c5**	12.99	22.38	48.74
**c6**	9.17	27.33	54.56

**Table 5:** Average number of MCP tool invocations per prompt category.

Category	gpt-5.2	gpt-5-mini	gpt-5-nano
**c1**	1.0	1.0	3.0
**c2**	2.2	2.6	3.0
**c3**	2.0	2.0	2.4
**c4**	2.0	2.4	2.2
**c5**	5.0	4.0	9.0
**c6**	3.2	4.8	7.6

**Table 6:** Number of correct responses per prompt category.

	gpt-5.2	gpt-5.2 No Instructions
**c1**	5/5	4/5
**c2**	5/5	3/5
**c3**	5/5	5/5
**c4**	5/5	3/5
**c5**	5/5	5/5
**c6**	4/5	4/5

## Data Availability

The original data presented in the study are freely accessible at https://github.com/iot-data-space/mcp.

## Appendix A. Prompts Used to Describe MCP Tools

### Appendix A.1. MCP Server Instructions

The following text is used for describing how an MCP server can be used:

*You are interacting with a data space that stores objects of multiple types. Each object has attributes relevant to its type, including a special attribute ‘located_in’ indicates its location. Users may ask about attributes for specific objects, types, or locations. If the user asks about a specific object, call read with object_id. If the user asks about a specific type, call read with type_id. If the user asks about a location, use get_types to discover which types contain the requested attribute or type description, then read by type_id and filter with located_in.*

### Appendix A.2. read() Tool Description

The following text is used for describing the read() tool to the MCP host:

*Read a specific object or all objects of a specific type. Provide a type identifier to fetch all objects of that type, or an object identifier to fetch a single object. Optionally pass filters to narrow results by attribute values, and optionally list which attributes to include in the response. Leave attributes empty to return all fields.*

Furthermore, for each input parameter, the following description is provided:

- type_id: The type identifier to filter objects by type
- object_id: The object identifier to fetch a specific object
- attributes: List of attribute names to include in the result
- filters: List of filter strings in the form ["attribute operator value", …]. Examples: ["temperature>30", "located_in==building1", "consumption<=20"]. Operators: ==, !=, <, <=, >, >=

### Appendix A.3. get_type() Tool Description

The following text is used for describing the get_type() tool to the MCP host:

*Retrieve matching types (including their attributes) from the data space by searching type and attribute descriptions for the provided keywords. Supply a comma-separated list of keywords; matching is case-insensitive and returns full type objects.* Furthermore, for its input parameter, the following description is provided:

- keywords: Comma-separated keywords to match against type and attribute descriptions (case-insensitive).

## Appendix B. Prompts Used for Evaluating the Implementation

Category 1:
  - Output only the number for the consumption reading of urn:mcp:plug1
  - Output only the number for the consumption reading of urn:mcp:plug2
  - Output only the number for the temperature reading of urn:mcp:thermometer4
  - Output only the number for the illuminance reading of urn:mcp:light_sensor3
  - Output only the number for the humidity reading of urn:mcp:humidity_sensor6
Category 2:
  - Output only the number for the average temperature in urn:mcp:building1
  - Output only the number for the average humidity in urn:mcp:building2
  - Output only the number for the average illuminance in urn:mcp:building4
  - Output only the number for the average power consumption in urn:mcp:building5
  - Output only the number for the average CO_2_ in urn:mcp:building3
Category 3:
  - Output only the number for the sum of consumption of all plugs
  - Output only the number for the sum of temperature of all thermometers
  - Output only the number for the sum of illuminance of all light sensors
  - Output only the number for the sum of humidity of all humidity sensors
  - Output only the number for the sum of CO_2_ of all air quality sensors
Category 4:
  - Output only the number for the sum of consumption of all plgs
  - Output only the number for the sum of temperature of all thermomters
  - Output only the number for the sum of illuminance of all lihgt sensors
  - Output only the number for the sum of humiity of all humedity sensors
  - Output only the number for the sum of CO_2_ of all air qualety sensors
Category 5:
  - Output only the number for the average HVAC setpoint in buildings where consumption is grater than 0.8
  - Output only the number for the sum of consumption of all plugs in buildings with postal-code 163 42
  - Output only the number for the average humidity in buildings with address containing Avanton
  - Output only the number for the average illuminance in buildings where door locks are unlocked
  - Output only the number for the sum of water consumption in buildings with at least one plug on
Category 6:
  - Output only the number for the average temperature of buildings located in Chalkida Greece
  - Output only the number for the average temperature in buildings located in Troias street
  - Output the name of the street with the hottest building
  - Output only the name of the greek city with the most buildings
  - One of the buildings of the dataset has water leakage. Output only its identifier
